# Supplementary material for: A cortical information bottleneck during decision-making
Source: bioRxiv. 2023 Jul 14:2023.07.12.548742. Preprint. [Version 1] doi: 10.1101/2023.07.12.548742 (PMC10369960; doi:10.1101/2023.07.12.548742)
Supplement: Supplement 1 [file NIHPP2023.07.12.548742v1-supplement-1.pdf]

bioRxiv preprint doi: https://doi.org/10.1101/2023.07.12.548742; this version posted July 14, 2023. The copyright holder for this preprint (which was not certified by peer review) is the author/funder, who has granted bioRxiv a license to display the preprint in perpetuity. It is made available under aCC-BY 4.0 International license.

Supplementary Information for: "A cortical information bottleneck during decision-making"

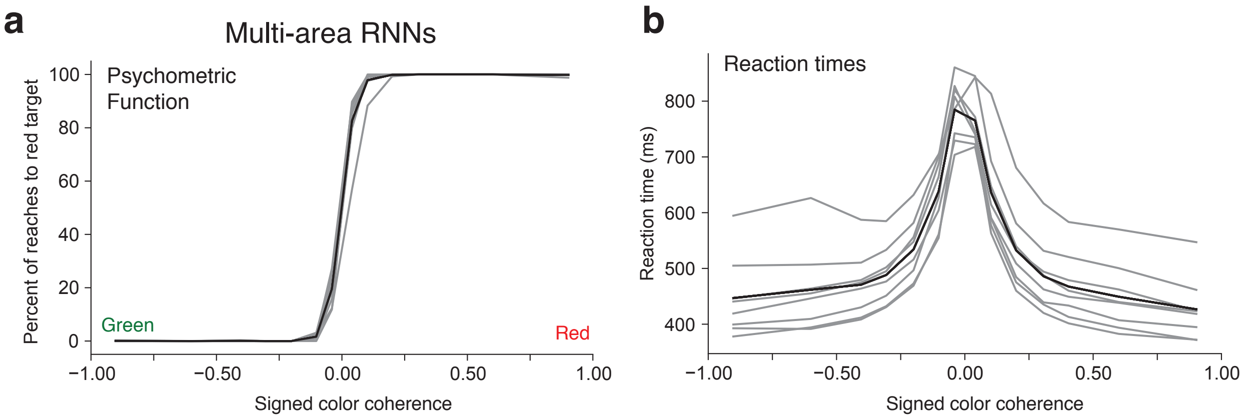

Figure S1: **(a)** Psychometric and **(b)** reaction time curves for multi-area RNNs. The hyperparameters used for these RNNs are described in Table 1. Gray lines represent individual RNNs and the black solid line is the average across all RNNs.

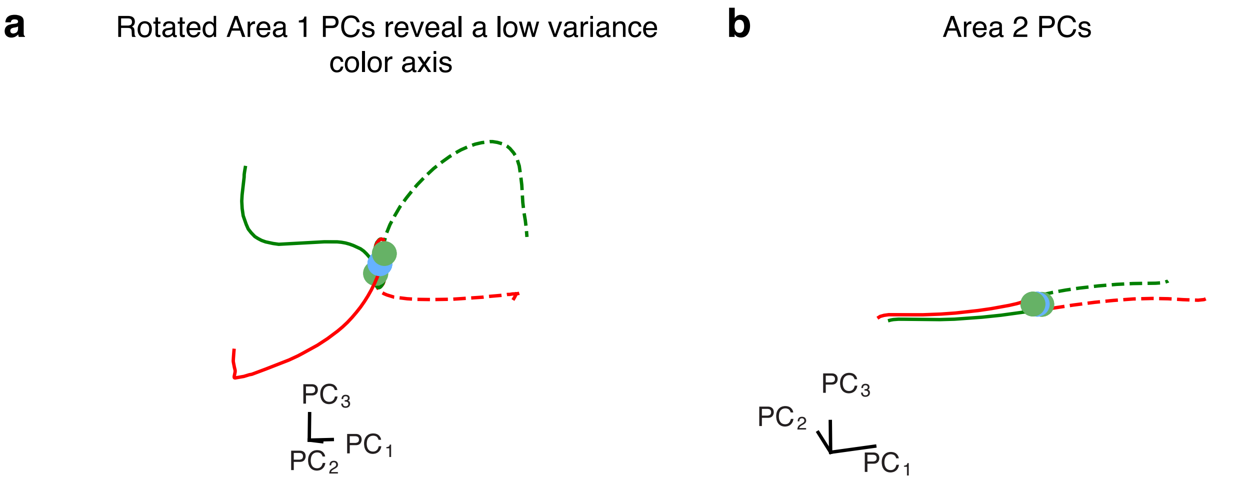

Figure S2: **(a)** Another rotation of the first three PCs for Area 1 RNN, with PC_3 amplified to show that there is a low variance color axis. **(b)** Area 2 PCs in the same projection as used in Figure 3. While these PCs qualitatively appear to represent the direction decision, they are distinct from Area 3, with Area 3 demonstrating a stronger resemblance to PMd activity.

bioRxiv preprint doi: https://doi.org/10.1101/2023.07.12.548742; this version posted July 14, 2023. The copyright holder for this preprint (which was not certified by peer review) is the author/funder, who has granted bioRxiv a license to display the preprint in perpetuity. It is made available under aCC-BY 4.0 International license.

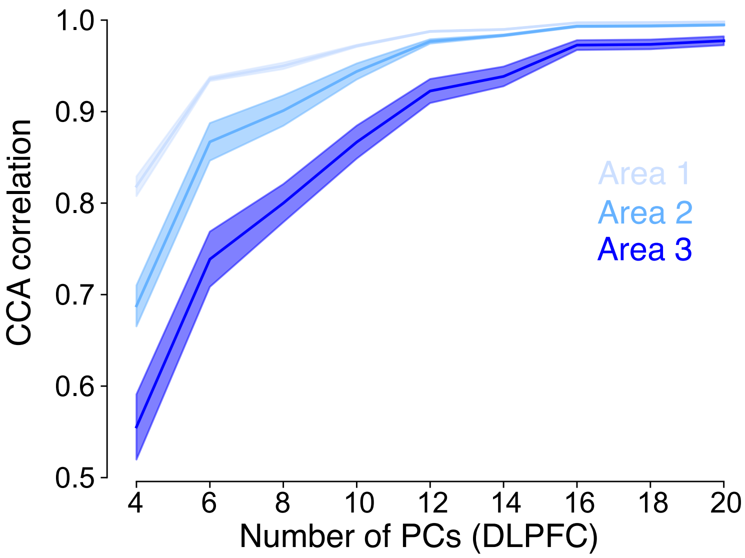

Figure S3: Because DLPFC is higher-dimensional than PMd, we performed the CCA correlation coefficient comparison to Areas 1-3 of the RNN varying the number of dimensions used for the DLPFC PCs. Note that as dimensionality increases, CCA correlation coefficient increases because additional dimensions, which are low variance, can be weighted to better reproduce the RNN PCs. We nevertheless observe that Area 1 has the highest CCA correlation to DLPFC, while Area 3 has the least.

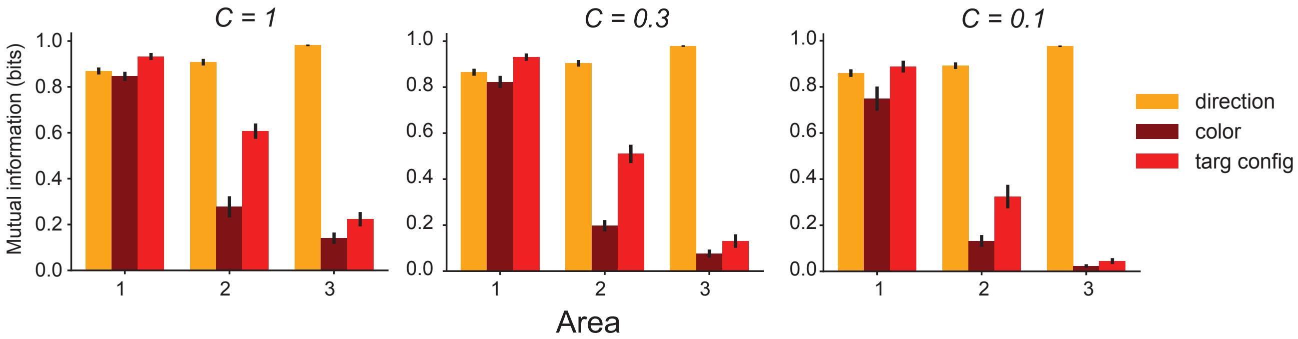

Figure S4: SVM Mutual Information (approximated using the Usable Information) for each RNN area as a function of increasing decoder regularization C. A lower C implies more regularization.

bioRxiv preprint doi: https://doi.org/10.1101/2023.07.12.548742; this version posted July 14, 2023. The copyright holder for this preprint (which was not certified by peer review) is the author/funder, who has granted bioRxiv a license to display the preprint in perpetuity. It is made available under aCC-BY 4.0 International license.

Intra-area dynamics separate direction and color

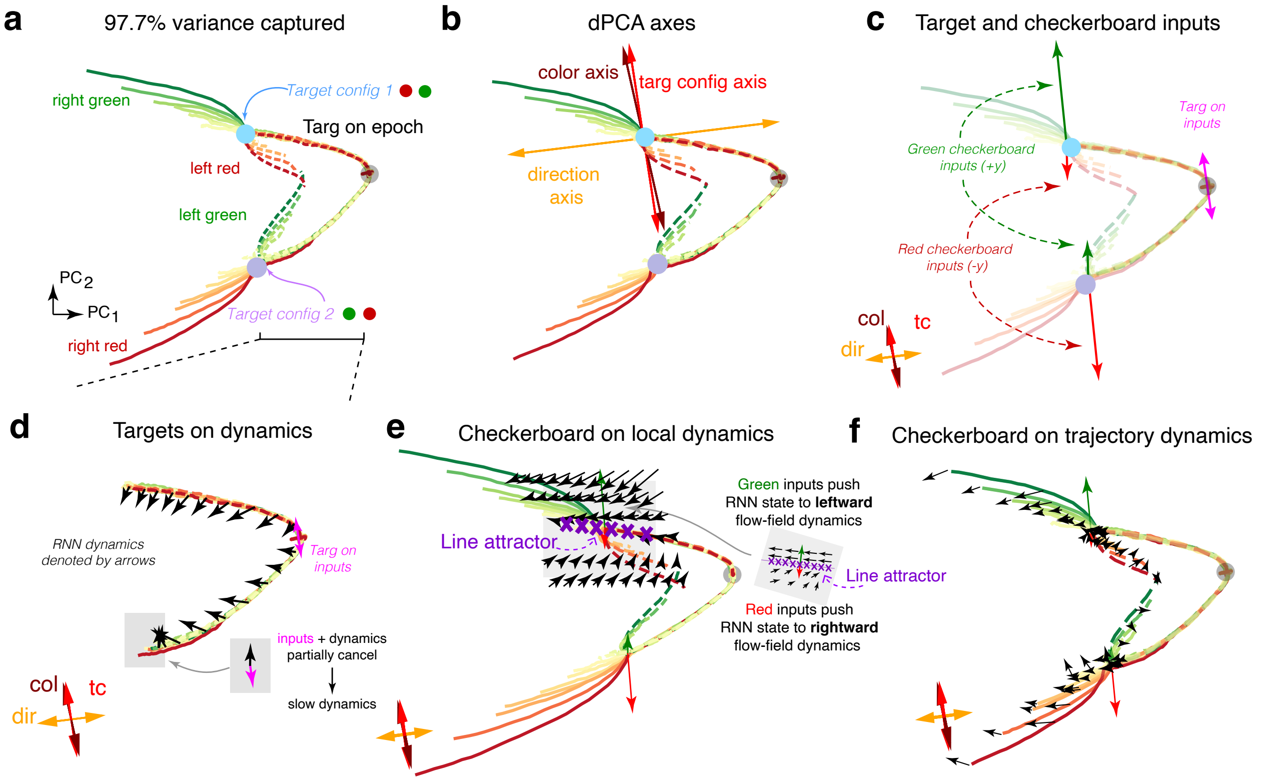

Figure S5: Candidate mechanism for axis orthogonalization. **(a)** Top 2 PCs of RNN Area 1 activity. Trajectories are now colored based on the coherence of the checkerboard, and the condition-independent signal is not removed. We did not remove the condition-independent signal so we could directly study the high-dimensional dynamics of the RNN and its equilibrium states. The trajectories separate to two regions corresponding to the two potential target configurations (Target config 1 in blue, Target config 2 in purple). The trajectories then separate upon checkerboard color input, leading to four trajectory motifs. **(b)** Projection of the dPCA principal axes onto the PCs. **(c)** Projection of the target configuration and color inputs onto the PCs. Target configuration inputs are shown in pink, a strongly green checkerboard in green, and a strongly red checkerboard in red. Irrespective of the target configuration, green checkerboards cause the RNN state to increase along PC_2 while red checkerboards cause the RNN state to decrease along PC_2. The strength of the input representation is state-dependent: checkerboards corresponding to left reaches, whether they are green or red, cause smaller movements of the RNN state along the color axis. **(d)** Visualization of RNN dynamics and inputs during the target presentation. In the Targets On epoch, target configuration inputs cause movement along the vertical target configuration axis. The RNN dynamics implemented a leftward flow-field that pushed the RNN state into an attractor region of slow dynamics. **(e)** At the Target config 1 attractor, we plot the local dynamics using a previously described technique[51]. The RNN implements approximately opposing flow fields above and below a line attractor. Above the attractor, a leftward flow-field increases direction axis activity, while below the attractor, a rightward flow-field decreases direction axis activity. A green checkerboard input therefore pushes the RNN state into the leftward flow-field (solid green trajectories) while a red checkerboard input pushes the RNN state into a rightward flow-field (dotted red trajectories). This computes the direction choice in a given target configuration, while allowing the direction axis to be orthogonal to color inputs. Arrows are not to scale; checkerboard inputs have been amplified to be visible. **(f)** Visualized dynamics across multiple trajectory motifs. These dynamics hold in both target configurations leading to separation of right and left decisions on the direction axis. Arrows are not to scale, for visualization purposes.

bioRxiv preprint doi: https://doi.org/10.1101/2023.07.12.548742; this version posted July 14, 2023. The copyright holder for this preprint (which was not certified by peer review) is the author/funder, who has granted bioRxiv a license to display the preprint in perpetuity. It is made available under aCC-BY 4.0 International license.

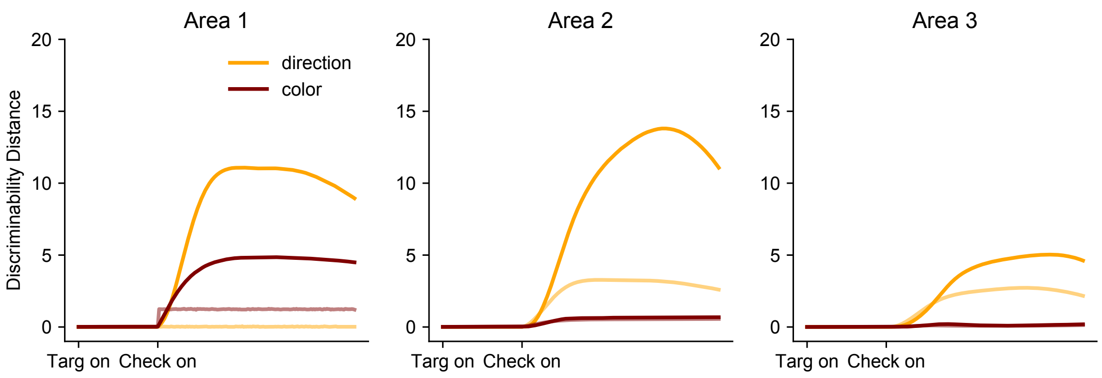

Figure S6: The norm of the direction discriminability (left red - right red + left green - right green)/2 and color discriminability (left green - left red + right green - right red)/2 as a function of the processing area. The inputs are shown in lighter transparency and the overall activity is shown in solid lines. Area 1 has significant recurrence evidenced by a large separation between the input and overall activity. For our exemplar network, there is very little evidence of recurrent filtering of color information (i.e recurrent activity is never below inputs).

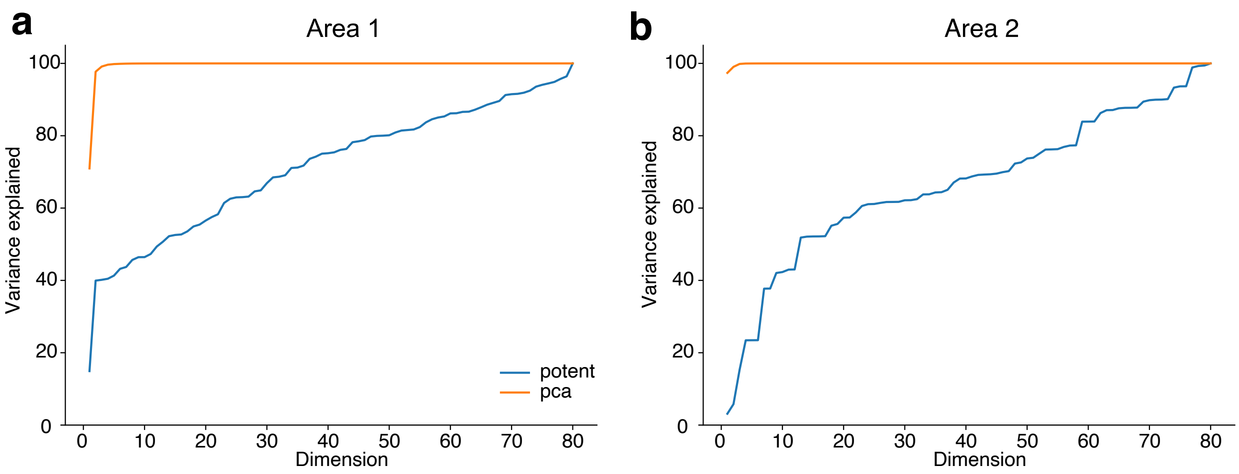

Figure S7: Relationship between PCs and inter-area potent space. (a) Variance explained of the excitatory units in Area 1 by the top principal components and top dimensions of potent space of $\mathbf{W}_{21}$, swept across all dimensions. **(b)** Variance explained of the excitatory units in Area 2 by the top principal components and top dimensions of potent space of $\mathbf{W}_{32}$, swept across all dimensions. These plots show that the connections between areas do not necessarily propagate the most dominant axes of variability in the source area to the downstream area. Excitatory units were used for the comparison because only excitatory units are read out by subsequent areas. These results were upheld when comparing to the variance explained by the top principal components obtained from all units.

bioRxiv preprint doi: https://doi.org/10.1101/2023.07.12.548742; this version posted July 14, 2023. The copyright holder for this preprint (which was not certified by peer review) is the author/funder, who has granted bioRxiv a license to display the preprint in perpetuity. It is made available under aCC-BY 4.0 International license.

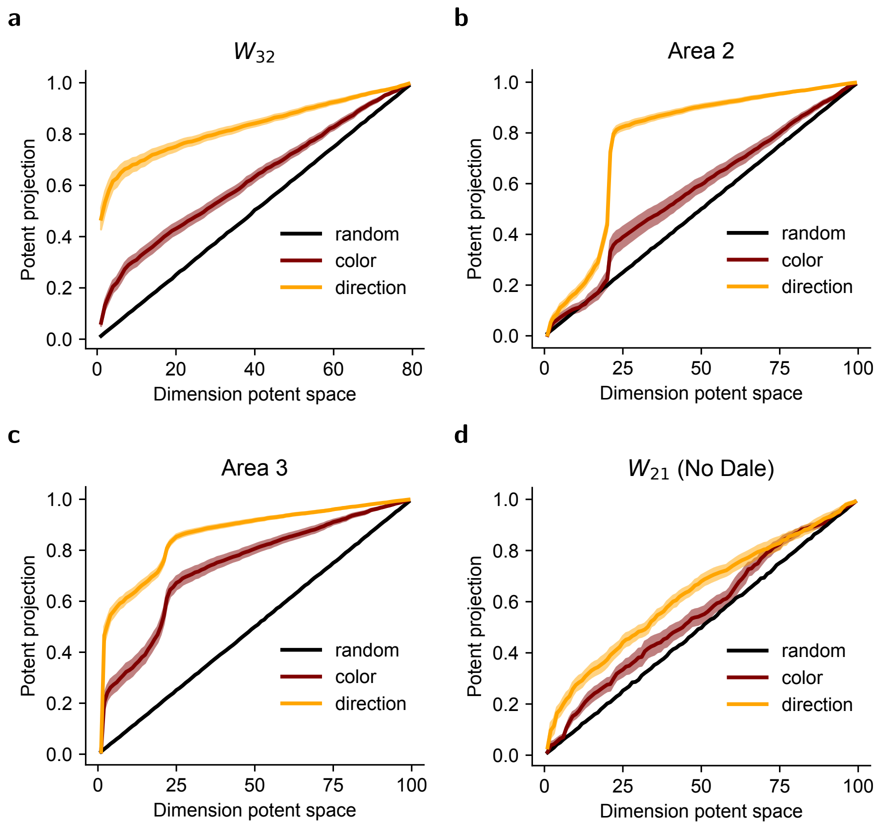

Figure S8: **(a)** Alignment of dpca color and direction axes from area 2 with inter-areal connections $\mathbf{W}_{32}$. **((b,c)** Alignment of dpca axes with intra-areal recurrent matrices for 3 area dale networks (Area 2 and Area 3). **(d).** Alignment of dpca axes in area 1 with $\mathbf{W}_{21}$ for networks without Dale's law. In contrast to Fig. 4f, direction information is not preferentially propagated. Same conventions as Fig. 4c,f.

bioRxiv preprint doi: https://doi.org/10.1101/2023.07.12.548742; this version posted July 14, 2023. The copyright holder for this preprint (which was not certified by peer review) is the author/funder, who has granted bioRxiv a license to display the preprint in perpetuity. It is made available under aCC-BY 4.0 International license.

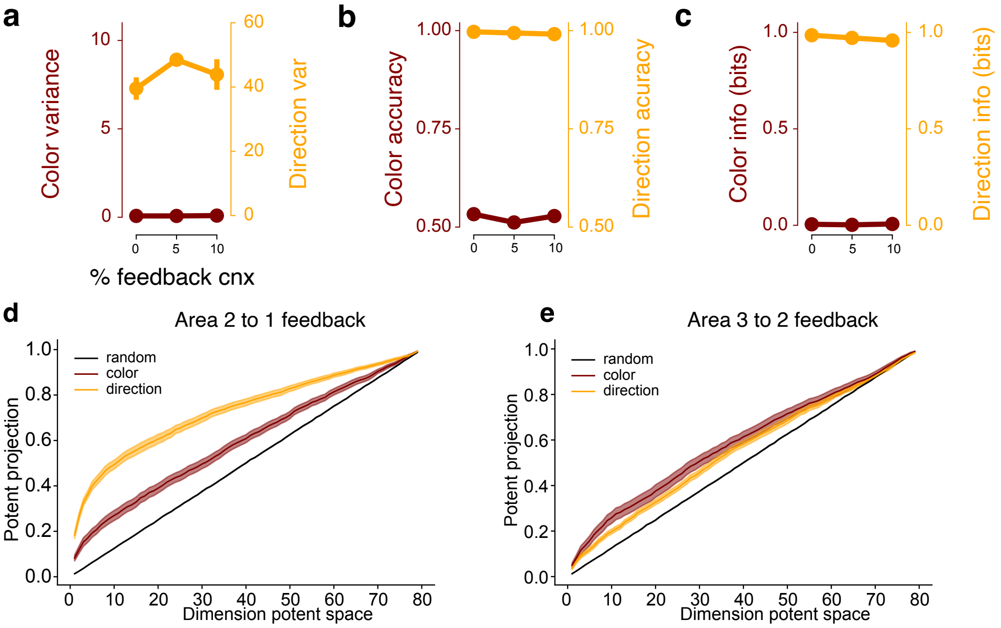

Figure S9: Effect of feedback connections (a) dPCA variance in area 3 of RNNs where we varied the amount of feedback connectivity. RNNs exhibited nearly zero dPCA color variance in Area 3 across networks with 0%, 5%, and 10% feedback connections. **(b, c)** RNNs also exhibited minimal color representations, achieving nearly chance levels of decode accuracy and nearly zero mutual information. **(d, e)** Feedback projections of the color and direction axis on the feedback inter-area matrix between **(d)** area 2 and area 1, and **(e)** area 3 and area 2 (for networks trained with 5% feedback connections, across variable feedforward connectivity percentages).

bioRxiv preprint doi: https://doi.org/10.1101/2023.07.12.548742; this version posted July 14, 2023. The copyright holder for this preprint (which was not certified by peer review) is the author/funder, who has granted bioRxiv a license to display the preprint in perpetuity. It is made available under aCC-BY 4.0 International license.

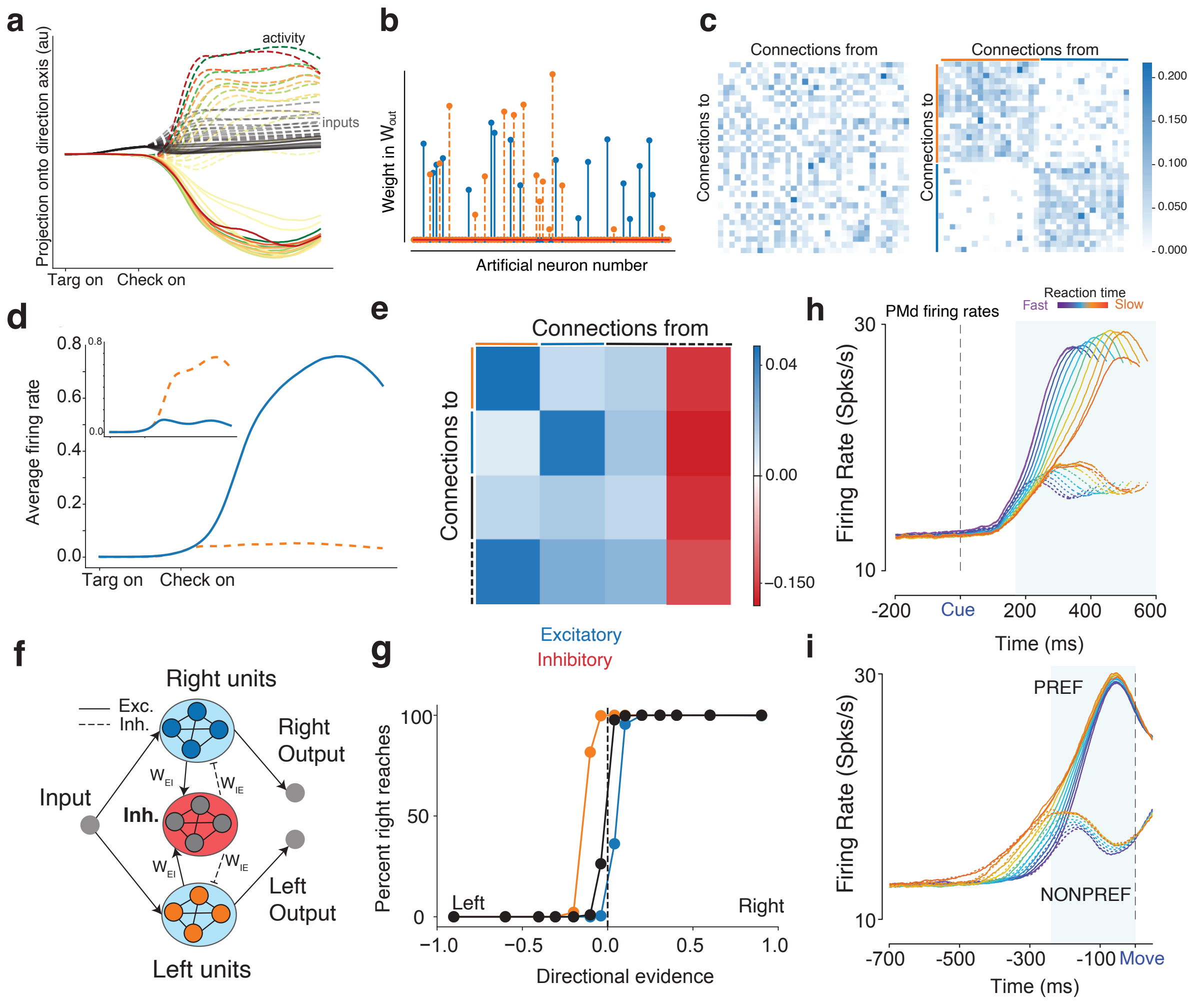

Figure S10: Area 3 mechanism. (a) Projection of input and overall activity onto the direction axis identified through dPCA. **(b)** Readout weights in $\mathbf{W}_{\mathrm{out}}$ are sparse, with many zero entries, and selective weights for a left or right reach. **(c)** The unsorted connectivity matrix for the nonzero readout units (left panel), and the sorted connectivity matrix when the matrix was reordered based on the readout weight pools (right). **(d)** Average PSTHs from units for a leftward reach and (inset) rightwards reach. When one pool increases activity, the other pool decreases activity. **(e)** Averaged recurrent connectivity matrix. **(f)** Schematic of output area. **(g)** Psychometric curve after perturbation experiment, where 10% of inhibitory weights to the left pool (orange) and right pool (blue) were increased (doubled). Directional evidence is computed by using the signed coherence and using target configuration to identify the strength of evidence for a left reach and strength of evidence for a right reach. Increasing inhibition to the left excitatory pool leads to more right choices and vice versa. **(h,i)** Firing rates of PMd neurons for PREF direction reaches and NONPREF direction reaches aligned to checkerboard and movement onset. In Winner-take-all models, when one pool wins the firing rate of the other pool is suppressed due to lateral inhibition. In PMd, when the PREF direction wins the firing rate of the NONPREF direction decreases (blue shaded region in i).

bioRxiv preprint doi: https://doi.org/10.1101/2023.07.12.548742; this version posted July 14, 2023. The copyright holder for this preprint (which was not certified by peer review) is the author/funder, who has granted bioRxiv a license to display the preprint in perpetuity. It is made available under aCC-BY 4.0 International license.

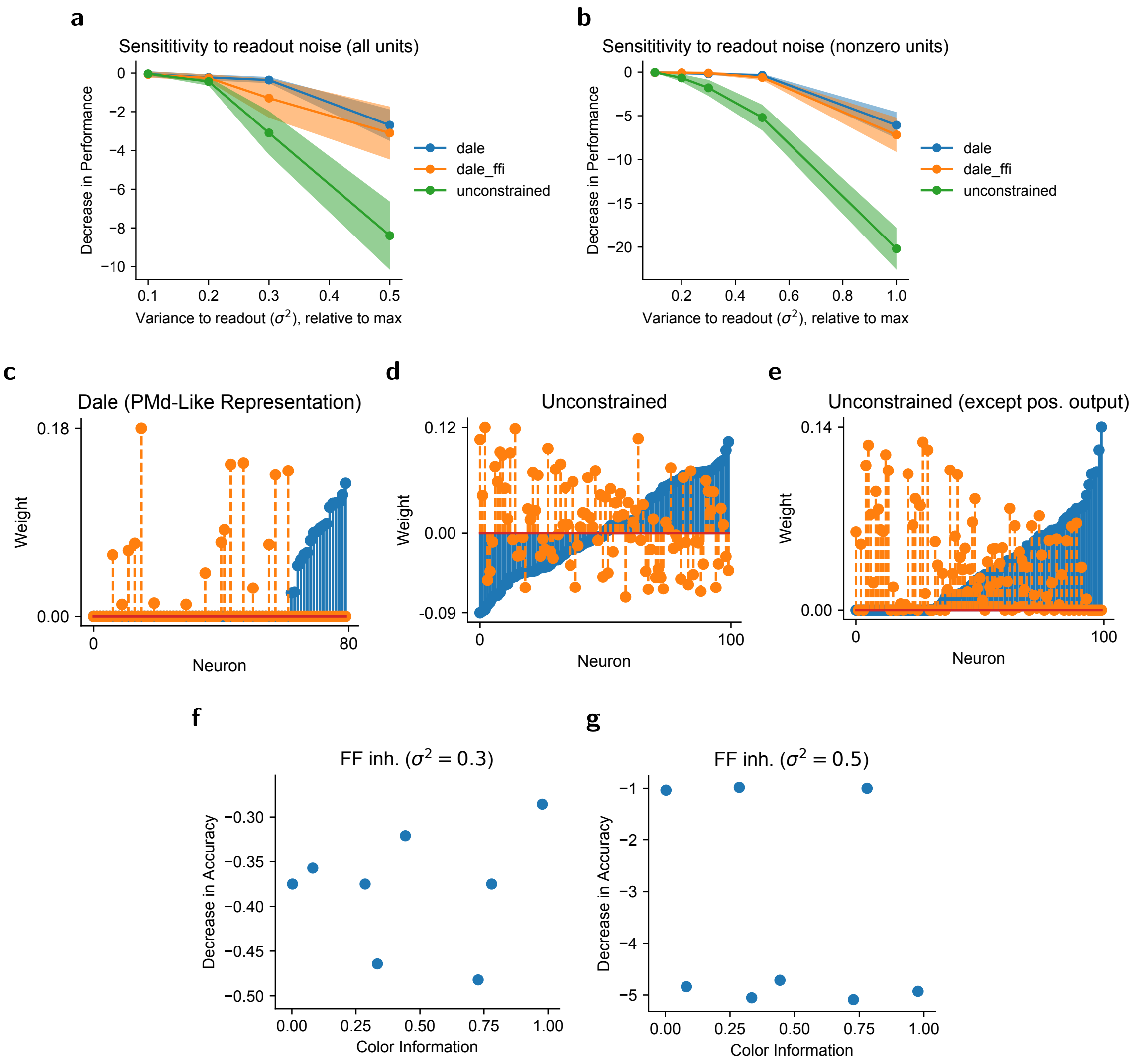

Figure S11: Potential multi-area computational advantage . **(Top Row)** Sensitivity to isotropic readout noise added to the output weights. **(a)** Noise added to all units in output (even the zero weights). **(b)** Noise only added to nonzero units. **(Middle Row)** Readout weights for left (dashed orange) and right (blue) reaches. **(c)** Readout weight with Dale's Law enforced, (**d**) Readout weights in unconstrained networks. (**e**) Readout weights in unconstrained but ensuring positive outputs. **(Bottom Row)** No correlation between robustness to noise and usable color information across random initializations for networks with 10% feedforward inhibition, where after training some networks had color information (Fig. 5b). We used a noise perturbation to each unit of variance **(f)** $\sigma^2 = 0.3$ and **(g)** $\sigma^2 = 0.5$.

bioRxiv preprint doi: https://doi.org/10.1101/2023.07.12.548742; this version posted July 14, 2023. The copyright holder for this preprint (which was not certified by peer review) is the author/funder, who has granted bioRxiv a license to display the preprint in perpetuity. It is made available under aCC-BY 4.0 International license.

Supplementary Note: Mutual Information Estimation

The entropy of a distribution is defined as

$$H(x) = \mathbb{E}_{x\sim p(x)}\left[\log \frac{1}{p(x)}\right]. \tag{8}$$

The mutual information, $I(X;Y)$, can be written in terms on an entropy term and as conditional entropy term:

$$I(Z;Y) = H(Y) - H(Y|Z). \tag{9}$$

We want to show that the usable information lower bounds the mutual information:

$$I(Z;Y) \geq I_u(Z;Y) := H(Y) - L_{CE}(p(y|z), q(y|z)) \tag{10}$$

It suffices to show that:

$$H(Y|Z) \leq L_{CE} \tag{11}$$

where L_{CE} is the cross-entropy loss on the test set. For our study, $H(Y)$ represented the known distribution of output classes, which in our case were equiprobable.

$$H(Y|Z) := \mathbb{E}_{(z,y)\sim p(z,y)}\left[\log \frac{1}{p(y|z)}\right] \tag{12}$$

$$= \underbrace{\mathbb{E}_{(z,y)\sim p(z,y)}\left[\log \frac{1}{q(y|z)}\right]}_{\text{cross-entropy loss}} - \underbrace{\mathbb{E}_{z\sim p(z)}\left[\mathrm{KL}(p(y|z)||q(y|z)\right]}_{\geq 0}, \tag{13}$$

$$\leq \mathbb{E}_{(z,y)\sim p(z,y)}\left[\log \frac{1}{q(y|z)}\right] := L_{CE} \tag{14}$$

To approximate $H(Y|Z)$, we first trained a neural network with cross-entropy loss to predict the output, Y, given the hidden activations, Z, learning a distribution $q(y|z)$. The KL denotes the Kullback-Liebler divergence. We multiplied (and divided) by an arbitrary variational distribution, $q(y|z)$, in the logarithm of equation 12, leading to equation 13. The first term in equation 13 is the cross-entropy loss commonly used for training neural networks. The second term is a KL divergence, and is therefore non-negative. In our approximator, the distribution, $q(y|x)$, is parametrized by a neural network. When the distribution $q(y|z) = p(y|z)$, our variational approximation of $H(Y|Z)$, and hence approximation of $I(Z;Y)$ is exact[20,52,53].

In the paper, we additionally report the accuracy of the neural network on the test set. This differs from the cross-entropy in that the cross-entropy incorporates a weighted measure of the accuracy based on how "certain" the network is, while the accuracy does not.
